# Prehabilitation Exercise Training to Target Improved Muscle Strength in Pretransplant Patients Diagnosed With Multiple Myeloma: Protocol for a Pilot Randomized Controlled Trial

**DOI:** 10.2196/64905

**Published:** 2024-12-19

**Authors:** Amber J Normann, Clifton C Mo, Rebekah L Wilson, Michelle Perez, Corey Cutler, Hajime Uno, LaDora V Thompson, Tina L Skinner, Paul G Richardson, Catherine R Marinac, Christina M Dieli-Conwright

**Affiliations:** 1 Department of Health Sciences Sargent College of Health & Rehabilitation Sciences Boston University Boston, MA United States; 2 Division of Population Sciences Department of Medical Oncology Dana-Farber Cancer Institute Boston, MA United States; 3 Jerome Lipper Center for Multiple Myeloma Research Department of Medical Oncology Dana-Farber Cancer Institute Boston, MA United States; 4 Harvard Medical School Boston, MA United States; 5 Division of Transplantation and Cellular Therapy Department of Medical Oncology Dana-Farber Cancer Institute Boston, MA United States; 6 Department of Data Science Dana-Farber Cancer Institute Boston, MA United States; 7 Department of Physical Therapy Boston University Boston, MA United States; 8 School of Human Movement and Nutrition Sciences The University of Queensland Brisbane Australia; 9 Dana-Farber Cancer Institute Boston, MA United States

**Keywords:** multiple myeloma, stem cell transplantation, exercise training, aerobic exercise, resistance exercise, preoperative exercise, muscle strength, physical fitness

## Abstract

**Background:**

Muscle mass and strength are severely compromised in patients diagnosed with multiple myeloma, such that the risk of poor overall survival increases as the prevalence of low muscle mass, also known as sarcopenia, increases. Additionally, at the time of autologous stem cell transplant (ASCT), 51% of patients experience low muscle mass and strength, which can prolong hospitalization and lead to increased risk of obesity, insulin resistance, lowered physical function, and poor quality of life.

**Objective:**

The PROTECT (Prehabilitation Exercise Training in Multiple Myeloma Patients Undergoing Autologous Stem Cell Transplantation) trial will examine the preliminary effects of digitally supervised prehabilitative aerobic and resistance exercise on muscle strength in patients with multiple myeloma scheduled for ASCT.

**Methods:**

This prospective, 2-armed single-center randomized controlled trial will recruit 30 patients with multiple myeloma, aged 18 years and older, planning to receive ASCT. Individuals will be assigned to either the exercise or the waitlist control group. The 8-week exercise intervention is home-based and digitally supervised by a clinical exercise trainer. The frequency of the exercise intervention is 3 times per week consisting of aerobic exercise on a cycle ergometer and resistance exercises, which are individually tailored based on patient health status. The waitlist control group maintains normal daily activities of living and is offered the intervention within 6 months from ASCT. The primary outcome is lower limb muscle strength, measured using the 10-repetition maximum leg press or extensor strength. Additional outcomes include physical and cardiorespiratory function, patient-reported outcomes, cardiometabolic health outcomes, and clinical outcomes.

**Results:**

The trial was funded in the fall of 2022 and recruitment began in June 2023. As of August 2024, a total of 3 participants have consented and been randomized (n=1, exercise group; n=2, waitlist control group). Trial completion and start of data analysis is expected in July 2025 with expected results to be published in early winter of 2026.

**Conclusions:**

We expect exercise to improve lower limb muscle strength and overall health outcomes compared to the waitlist control group. Results will contribute foundational knowledge needed to conduct larger-phase clinical trials testing the clinical benefits of prehabilitation exercise in this patient population. This study will provide insight into a prehabilitative exercise intervention designed to support patient prognosis.

**Trial Registration:**

ClinicalTrials.gov NCT05706766; https://clinicaltrials.gov/study/NCT05706766

**International Registered Report Identifier (IRRID):**

DERR1-10.2196/64905

## Introduction

### Background

Across diseases with transplant-eligible patients, multiple myeloma diagnoses make up 35% of autologous stem cell transplants (ASCT) [1,2]. Due to advancements in this routine noncurative therapy that replaces diseased bone marrow with healthy blood stem cells, life expectancy has been extended by an estimated 5-10 years in patients with multiple myeloma [3]. However, patients with multiple myeloma who receive ASCT often experience physical deconditioning prior to ASCT [4], which leads to a reduced ability in activities of daily function [5] that may contribute to loss of muscle mass and strength. Lowered skeletal muscle mass, a characteristic of sarcopenia, prior to ASCT is correlated with postoperative complications [6]. Patients receiving ASCT, who can experience sarcopenia or sarcopenic obesity at the time of transplant, have an increased risk of worsened overall survival [7].

Patients with multiple myeloma who participate in the ~150 minutes of exercise, as recommended by the American College of Sports Medicine [8,9], experience better clinical outcomes including revised myeloma comorbidity index scores, treatment tolerance, length of hospital stay, remission status, overall survival, and progression-free survival compared to age-matched inactive patients [10]. Physical fitness outcomes, such as muscle strength and cardiovascular fitness, along with quality of life and fatigue, are improved in patients with multiple myeloma who undergo aerobic and resistance exercise training [11]. Previous trials have shown progressive aerobic and resistance exercise training as safe with no adverse events [12], particularly when implemented post-ASCT in patients with multiple myeloma [11,13]. Moreover, preliminary feasibility data indicates challenges with in-person attendance during the pre-ASCT phase and presents the opportunity to study a digitally supervised prehabilitation model to support already suppressed activity levels in this population [14]. With the intent to optimize muscle strength and physical fitness, precise exercise prescription (ie, intensity, duration, and frequency) is currently unclear, given the different exercise program modalities used in patients with multiple myeloma over the past 2 decades [12,13,15-20].

### Objectives

**Figure 1:**
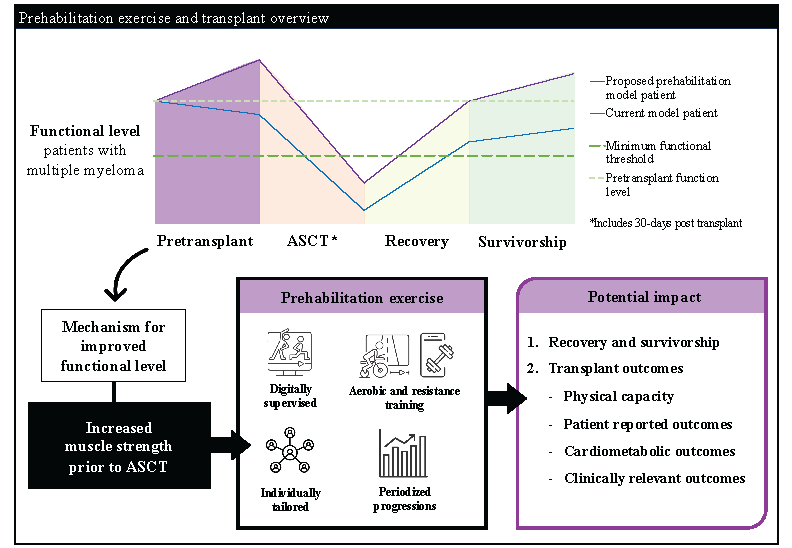
Conceptual framework of the PROTECT Trial. ASCT: autologous stem cell transplant; PROTECT: Prehabilitation Exercise Training in Multiple Myeloma Patients Undergoing Autologous Stem Cell Transplantation.

The goal of this randomized controlled trial, “Prehabilitation Exercise Training in Multiple Myeloma Patients Undergoing Autologous Stem Cell Transplantation: The PROTECT (Prehabilitation Exercise Training in Multiple Myeloma Patients Undergoing Autologous Stem Cell Transplantation) trial (Figure 1),” is to assess the impact of digitally supervised, individually tailored prehabilitative aerobic and resistance exercise in patients with multiple myeloma undergoing ASCT (Figure 1) on lower limb muscle strength (primary objective) in the exercise group compared to the waitlist control group. The secondary and tertiary objectives will examine physical capacity (physical function and cardiorespiratory fitness) and patient-reported outcomes (fatigue and quality of life), cardiometabolic health outcomes (biomarkers and body composition), along with posttransplant clinical outcomes including Hematopoietic Cell Transplantation-Comorbidity Index, length of hospital stay, readmission rates, and treatment-related adverse events. Finally, due to the nonprofound changes given the limited intervention period, the exploratory objective is to evaluate the exercise intervention completed post-ASCT on muscle strength, physical capacity, patient-reported outcomes, and cardiometabolic health outcomes. We hypothesize that prehabilitative aerobic and resistance exercise will improve lower limb muscle strength prior to receiving ASCT and 30 days posttransplant in the exercise group compared to the waitlist control group.

## Methods

### Study Design

**Figure 2:**
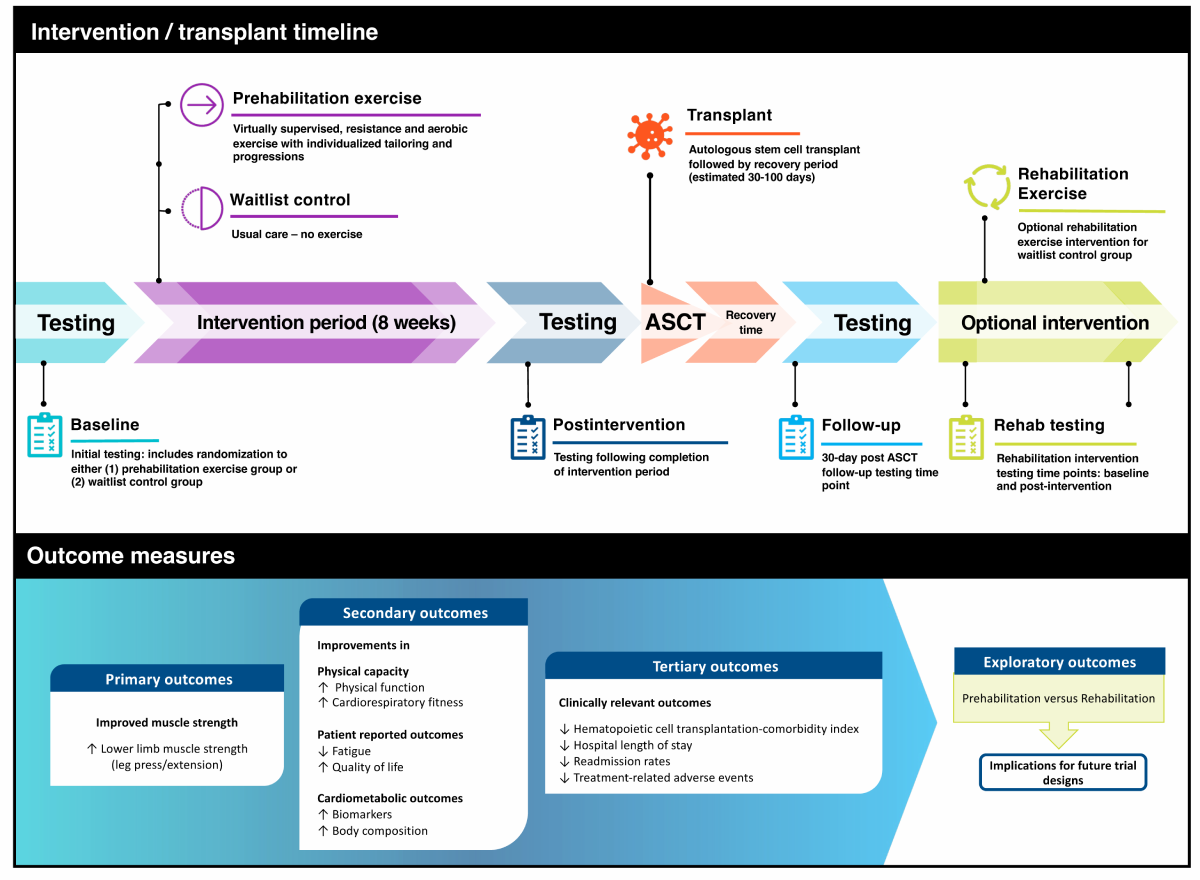
Study outline for the PROTECT Trial. ASCT: autologous stem cell transplant; PROTECT: Prehabilitation Exercise Training in Multiple Myeloma Patients Undergoing Autologous Stem Cell Transplantation.

The PROTECT trial is a prospective, single-center, two-arm randomized controlled trial conducted at the Dana-Farber Cancer Institute (Boston, Massachusetts). Patients with multiple myeloma scheduled to receive ASCT are randomly assigned to one of two groups: exercise group or waitlist control group. The study schema and timeline (Figure 2) involve an 8-week intervention with a 30-day posttransplant follow-up. This study protocol is in accordance with the Consolidated Standards of Reporting Trials guidelines [21].

### Participants and Recruitment

This trial includes patients diagnosed with multiple myeloma who are scheduled to receive ASCT with the full inclusion and exclusion criteria depicted in Textbox 1. Potential participants will be identified at the Dana-Farber Cancer Institute through various recruitment strategies including screening hematologic oncology clinic lists, and use of patient advertisements in waiting areas. A study coordinator contacts the patients’ treating providers or oncologists to request permission to contact potential participants who are identified through patient lists. Further screening of potentially eligible participants is conducted by study staff in person or by phone to confirm remaining eligibilities, which includes the use of the Godin Leisure-Time Exercise Questionnaire [22] to assess physical activity history and using questions similar to the Physical Activity Readiness Questionnaire to assess patient’s health status [23]. Patients interested in participating are scheduled for a digital or in-person visit to review the protocol and sign informed consent, with the option to sign consent electronically.

**Inclusion and exclusion criteria of the PROTECT Trial.**

**Inclusion criteria**

- Aged 18 years and older.
- All patients will have a diagnosis of multiple myeloma, referral to the Dana-Farber Cancer Institute transplant team, and assigned to the autologous transplantation waiting list for a first transplant.
- Planning to receive autologous stem cell transplant after 6 weeks with or without concurrent neoadjuvant treatments at Dana-Farber Cancer Institute.
- Medical clearance to perform moderate to vigorous intensity aerobic and resistance exercise intervention and fitness testing by their treating physician or a certified clinical exercise physiologist.
- Speak English.
- Currently participating in less than or equal to 60 minutes of structured moderate to vigorous intensity exercise per week.
- Willing to travel to Dana-Farber Cancer Institute for necessary data collection.
- Ability to understand and the willingness to sign a written informed consent document.
- The effects of exercise on the developing fetus are unknown. For this reason, women of child-bearing potential must agree to undergo a pregnancy test and to use adequate contraception (hormonal or barrier method of birth control or abstinence) prior to study entry and for 6 months following duration of study participation. Should a woman become pregnant or suspect that she is pregnant while participating in the trial, she should inform her treating physician immediately.

**Exclusion criteria**

- History of unstable angina, abnormal resting electrocardiogram or unstable angina, or a heart attack in the previous month to allow safe completion of the cardiopulmonary exercise test or VO_2_ (maximum rate of oxygen consumption) peak test.
- Patients with known spinal instability, spinal cord compression or neurological deficits, or contraindications that preclude exercise.
- Those who have had recent (within 6 weeks) spinal surgery or other intervention surgery or pathological fractures.
- Those deemed unsuitable to partake by the transplant or study team.
- Patients at high risk of impending pathologic fracture of a weight-bearing bone (including spine, hip or femur, and humerus) as determined by a physician.
- Unable or unwilling to undertake an exercise program on a regular basis.
- Pre-existing musculoskeletal or cardiorespiratory disease, or metabolic diseases that could exacerbate with exercise, in addition to other conditions deemed unsafe by physician.
- Patients with other active malignancies requiring active therapy.
- Participate in more than 60 minutes of structured moderate to vigorous intensity exercise per week.
- Unable to travel to Dana-Farber Cancer Institute for necessary data collection.
- Participants, who in the opinion of the investigator, may not be able to comply with the safety monitoring requirements of the study.

### Ethical Considerations

Approval of the trial protocol was obtained from the institutional review board at the Dana-Farber Cancer Institute (22-403) and registered at ClinicalTrials.gov (NCT05706766). All participants review and sign an informed consent document that includes the option to opt-out at any point during the trial. Per Dana-Farber Cancer Institute policy, all patient identification information is deidentified. Participants are provided compensation (US $25) for each testing visit completed in the form of a cash spending card.

### Randomization and Blinding

Participants are randomly assigned to either the exercise group or the waitlist control group in a 1:1 ratio following the completion of baseline assessments. Prior to the trial start, the study biostatistician prepared the randomization schema and provided the randomization allocation to staff through a web-based application (REDCap [Research Electronic Data Capture]; Vanderbilt University) [24] to blind study investigators to the randomization process. The nature of the exercise intervention prevents blinding the study participants, interventionists, and outcome assessors. However, study investigators adhere to a set protocol to standardize outcome assessments and avoid bias.

### Prehabilitative Aerobic and Resistance Exercise Intervention

#### Overview

The exercise group receives a prehabilitative, home-based, digitally supervised multimodal (aerobic and resistance) 8-week exercise program led by a clinical exercise trainer. The program consists of 24 sessions that are conducted three times per week for eight weeks, with each session approximating 60-90 minutes. Blood pressure will be assessed with an automatic blood pressure cuff prior to the start of each session and heart rate (HR) will be monitored using a real-time HR monitor (ie, Fitbit watch), both devices are provided to the participant. Each session includes a 5-minute warm-up of low-intensity aerobic exercise, prescribed as 57%-64% of HR maximum [25], and a cool-down stretching period with 3-4 static stretches that target major muscle groups, held for 30 seconds each. The aerobic intensity and resistance load for each exercise are determined through one-repetition maximum (1RM) and peak oxygen consumption, respectively, during baseline testing. A qualified exercise trainer will individually tailor training, with gradual progression based on current exercise guidelines and principles during the digitally supervised sessions [26].

Upon completion of baseline testing, patients randomized to the exercise group are provided an introductory session in-person for exercise familiarization delivered by a clinical exercise trainer that will include an overview of the exercise program and coach a reduced sample exercise session to ensure activation of target muscle groups in each exercise. The intervention systematically progressed (Table 1) from low intensity and gradually increased to high intensity over the 8-week duration based on patient health status and ability. Enrolled patients will be provided, at no cost, a Wi-Fi–enabled tablet for digital delivery of training, a stationary bike, and sets of adjustable resistance bands. Each session will include 5 resistance exercises with 1 core exercise, and up to 20 minutes of aerobic cycling.

**Table 1:** Progression model for the prehabilitative aerobic and resistance exercise intervention.

Intervention time point	Resistance exercise progression^a^				Aerobic exercise progression	
Intervention timepoint	Intensity (%1RM^b^)	Sets	Repetitions	Intensity (% HR^c^_max_)		Duration (minutes)
1	50	2	12-15	60		15
2	55	3	12-15	60		15
3	60	3	12-15	65		15
4	60	3	12-15	65		15
5	65	3	10-12	70		20
6	65	3	10-12	70		20
7	70	3	8-10	75		20
8	70	3	8-10	75		20

^a^Resistance exercises: up to 3 upper body, 3 lower body, and 1 core exercises will be selected. Depending on the daily needs of the participant and bone lesion site, alternative exercises may be prescribed at the discretion of the exercise trainer.

^b^1RM: one-repetition maximum.

^c^HR: heart rate.

#### Resistance Exercise

Resistance exercise will target upper and lower body muscle groups with each exercise performed as 2-3 sets of 8-15 repetitions at intensities 50%-75% 1RM. Resistance exercise training will be performed using body weight and progressed with elastic resistance bands of various strengths in accordance with individual patient health. Resistance training will start at 50% of the participant’s 1RM and increase in intensity every two weeks by 5% or 10% until the goal of 70% 1RM is reached. The number of repetitions will increase in order for participants to perform 3 sets of 8-10 repetitions by week 8. Rest periods of ~60 seconds will be counted between each set of resistance exercises. The clinical exercise trainer will make adjustments for patients who progress beyond the estimated weights from the 10RM testing or are unable to meet the prescribed intensities.

#### Aerobic Exercise

Prescribed aerobic exercise will target cardiovascular health and lower limb strength through 15- to 20-minute training bouts consisting of a 60%-75% HR maximum. Digitally supervised sessions are tailored by clinical exercise trainers using gradual progressions based on current exercise guidelines and principles [26]. Aerobic exercise will begin at 60% HR maximum and progress in an alternating fashion in either intensity or duration, by 5% or 5-minute increments, respectively, each week. Appropriate exercise intensity will be maintained using a HR monitor during each session.

#### Measures During Exercise Sessions

At the start of each training session, the clinical exercise trainer will use the Therapy-Related Symptom Checklist to assess and identify participant-reported symptom occurrence and severity in each exercise session [27]. The clinical exercise trainer will record participants verbal responses, at the start and end of each session, to the Exercise-Induced Feeling Inventory, which assesses an individual’s feeling state postexercise and is also used for the assessment of self-motivation to adhere to an exercise regimen [28].

#### Waitlist Control

The waitlist control group will receive usual care during the initial visit that includes counseling about the details of the surgery and anticipated recovery. Participants in the waitlist control group will be offered the PROTECT intervention and exercise resources within 6 months of the end of the study period (after the 30-day post-ASCT follow-up time point), based on the desired participant start date.

### Outcome Measures

#### Overview

The outcomes are assessed at baseline, postintervention, and 30-day posttransplant follow-up. All measures are performed by trained study personnel at the Dana-Farber Cancer Institute. Postintervention assessment (week 10; visit 27) includes a reassessment of baseline measures. The 30-day post-ASCT follow-up (week 14-15; visit 28) consists of a reassessment of baseline measures (not including dual-energy x-ray absorptiometry [DEXA]) in addition to Hematopoietic Cell Transplantation-Comorbidity Index, length of hospital stay, readmission rates, and treatment-related adverse events. If the intervention is selected following the ASCT recovery period, the waitlist control group testing (visits 29-54) includes assessment of baseline measures (not including DEXA) pre- and postintervention (visits 29 and 54, respectively).

#### Primary Outcomes—Lower Limb Muscle Strength

Percent change in lower limb muscle strength is the primary outcome. The 1RM value will be estimated from 10RM muscle strength tests on the leg press or leg extension. The test selected is dependent on the bone lesion site and the participant's health status. Additional strength testing using approximately 5 resistance band exercises individually selected for participants based on health status; 1RM values will be calculated and reported using validated equations [29,30]. Alternative strength exercises will be used to ensure participants’ safety as needed for both testing and training periods. Following a warm-up, 3-5 attempts will be given to reach the final 10RM load with a 2-minute rest period between attempts.

#### Secondary Outcomes

##### Physical Capacity

Physical capacity, in this study, is comprised of physical function measures (a total of five) and cardiorespiratory fitness.

Physical function is assessed by the Short Physical Performance Battery, a validated measure that includes 3 sections [31]. This includes the following three lower extremity measures, all held for 10 seconds or when the participant steps out of position, (1) timed balance is assessed through the side-by-side stand, semitandem stand, and tandem stand. (2) Gait speed is assessed through the time recorded over a 4-meter distance and participants are allowed two attempts. (3) Chair stand is performed through a single chair stand and five repeated chair stands in quick succession that are timed to completion.

Physical function is also assessed through the Margaria Stair Climb, Timed Up-and-Go test, Sit-to-Stand test, and handgrip strength. The Margaria Stair Climb measures functional power using a stair climb test that has been successfully performed and correlated with lower-extremity power and mobility performance in older adults [32]. Participants ascend a flight of 10 stairs one step at a time as quickly as possible without hand support and the average time from the third to the ninth stair, out of 3 trials, is calculated to the nearest 0.01 second. The Timed Up-and-Go test assesses mobility and has been shown to predict immediate fall risk better than static balance tests or isometric muscle strength [33]. Participants begin seated in a chair with hands on the armrests and are asked to rise, walk to a line on the floor 3 meters from the chair, turn around, and return to the same seated position as quickly and safely as possible. Scores are taken as the average time of three trials to complete the task. The Sit to Stand test includes participants rising to a full standing position and sitting back down repeatedly for 30 seconds, the total number of repetitions is recorded. Finally, grip strength is measured using a hand-held dynamometer on the participant’s dominant hand, where they grip the handle of the dynamometer with one hand with as much pressure as possible for three seconds.

Cardiorespiratory fitness (functional aerobic capacity) is assessed as VO_2peak_ (peak aerobic capacity) during exercise exertion testing on a stationary bike, where participants complete a maximal cycling protocol that comprises a 10W increase in workload every one minute, starting at 40W and concludes when the participant is unable to physically cycle.

Criteria for VO_2peak_ must meet two of the three following criteria: (1) participant reports the reason for stopping the test is due to dyspnea or muscular (leg) fatigue or a similar description indicating volitional fatigue or exhaustion as evaluated by the qualified exercise physiologist; (2) respiratory exchange ratio≥1.10; and (3) rating of perceived exertion (RPE)≥7 on the 0-10 Borg scale. During the test, gas exchange is measured by a metabolic cart (TrueOne 2400, ParvoMedic Inc, Salt Lake City, Utah) and VO_2max_ is determined as the highest oxygen value obtained at maximal exertion. HR (Polar USA, Lake Success, New York) and RPE (Borg CR10 scale) [34] are recorded during the test. If maximal testing is not permitted or unable to be performed, participants may complete the YMCA submaximal cycling protocol to measure estimated maximal aerobic capacity and maximal power output. The initial workload will be set at 150 kg/min and includes three-minute intervals. The predicted maximum workload and oxygen uptake will be determined using the plotted points of the HR versus workload (kg/m/min) and the participant’s estimated HR max (220-age) [35].

##### Participant Reported Outcomes

Various aspects of psychosocial outcomes are assessed using validated questionnaires, including the PROMIS Cancer Fatigue Short Form to assess fatigue [36,37], the Vulnerable Elders Survey-13 to predict functional decline and mortality [38,39], and the Clinical Frailty Scale, to evaluate, evaluates comorbidity, function, and cognition [40]. The Functional Assessment of Cancer Therapy–Multiple Myeloma is a 41-item measure for quality of life in patients with multiple myeloma [41]. The Functional Assessment of Cancer Therapy-Bone Pain assesses bone pain and its effect on the quality of life and changes in response to treatment [42]. The Brief Pain Inventory short version is a 2-factor questionnaire to assess location and pain severity using a visual diagram and has been validated in patients with cancer [43,44]. The Hospital Anxiety and Depression Scale is a self-rated scale to assess psychological distress [45]. Finally, sleep quality will be assessed using the Pittsburg Sleep Quality Index, which contains 19 questions evaluating 7 domains of sleep- subjective sleep quality, sleep latency, sleep duration, habitual sleep efficiency, sleep disturbances, use of sleep medication, and daytime dysfunction [46,47].

##### Cardiometabolic Outcomes

Fasting (12-hour fast) blood is drawn from the antecubital vein (~30 cc) by a trained phlebotomist. For each sample, a total of six 10 mL tubes will be collected, three ethylenediaminetetraacetic acid and three Serum tubes, along with one additional lavender-topped tube (whole blood in ethylenediaminetetraacetic acid), for a total of 62 mL of blood drawn at each testing timepoint. Samples are centrifuged and then aliquoted and stored at –80 °C for future measures. Blood samples will be transported to Brigham Research Assay Core near the study end for batch-testing analysis of glucose, high-density lipoprotein-cholesterol, low-density lipoprotein-cholesterol, insulin, insulin resistance (Homeostatic Model Assessment for Insulin Resistance), triglycerides, and glycosylated hemoglobin. Future measures will include the analysis of additional biomarkers.

Lean mass, fat mass, and body fat % are obtained from the whole-body DEXA scan (completed only at baseline and postintervention testing timepoints). Premenopausal participants complete a pregnancy test (urine sample) prior to the body composition assessment to ensure the participant is not pregnant. BMI in kg/m^2^ will be calculated from height and weight. A constant tension tape measure is used to obtain waist circumference defined as the distance around the waist using the umbilicus as the reference point. A constant tension tape measure is used to obtain hip circumference defined as the distance around the widest girth of the buttocks using the greater trochanter as a landmark. Body composition is assessed via bioelectrical impedance using a validated device (Tanita 780, Arlington Heights, Illinois). The device will estimate body fat using an algorithm based on age, sex, height, and body weight.

#### Tertiary Outcomes—Posttransplant Clinical Outcomes

Posttransplant clinical outcomes are assessed through a review of patient medical records and include the Hematopoietic Cell Transplantation-Comorbidity Index, length of hospital stay, readmission rates, and treatment-related adverse events.

#### Exploratory Outcomes

The treatment trajectory for patients with multiple myeloma incorporates prehabilitation and rehabilitation, though when a patient experiences the most benefit is unclear, as they occur at different stages of the treatment trajectory. Therefore, a self-controlled single-group analysis will be done on the participants from the waitlist control who elect the exercise program post-ASCT to assess how an 8-week exercise intervention completed post-ASCT affects muscle strength, physical capacity, patient-reported outcomes, and cardiometabolic health outcomes.

### Adverse Events

Participants are evaluated for potential adverse events (eg, pain, muscle soreness, nausea, and bone pain) during each exercise session in the study period. Phone numbers for study staff are provided to report potential intervention-related adverse events. The National Cancer Institute Common Terminology Criteria for Adverse Events V5 is used to assess participants and documented by the study staff at each exercise session. Study progress and injuries are reported upon during weekly meetings through in-person updates, phone calls, or weekly email updates. Adverse events, both serious and nonserious, and deaths encountered from initiation of study intervention, throughout the study, and within 30 days of the last study intervention sessions, are followed to resolution, or until the principal investigator determines the participant as stable or the event as irreversible, or the participant is lost to follow-up.

### Sample Size

We anticipate recruiting 1-2 participants per month, based on the accrual rates (3 to 4 participants per month) of previous exercise intervention studies [48]. A total of 30 eligible participants will be entered into this study and randomly assigned to the intervention and the control groups in a 1:1 ratio (15 per group). The sample size is based on general recommendations (24 to 50) for pilot studies in general [49-51] and this study provides 80% power to detect the effect size of Cohen *d*=1.15 (taking account of 10% dropout) at .05 two-sided α level.

### Statistical Analysis

The primary analysis is a mean difference in percent change from baseline (week 0, preintervention), between the intervention and the control groups in lower leg muscle strength over 2 time points: pretransplant (week 9, postintervention), and posttransplant (week 14-15, 30-day post-ASCT follow-up). Linear mixed-effects models are used to compare the between-group differences at postintervention and follow-up after adjusting for baseline value of the outcome and covariates, where the intervention indicator and time are included as fixed effects and participants are included as random effects. From previous studies, the estimated standard deviation of change in muscle strength (measured through leg press) from baseline to pretransplant is 16.8 kg. Thus, this study will detect a between-group mean difference of 19.3 kg with 80% power. The primary analyses will be based on the intention-to-treat principle including all randomized participants. The same analyses will also be performed with the per-protocol population.

To test hypotheses related to differences in physical capacity, patient-reported outcomes, and cardiometabolic health outcomes across groups at study completion, standard statistical techniques are used to assess within-group differences among each end point (ie, 2-tailed *t* test or Wilcoxon signed rank test, proportions, and corresponding 95% exact CI, etc) and between-group differences (ie, 2-tailed *t* tests, two-sample Wilcoxon tests, generalized linear mixed-effects model for longitudinal measurements, and so on). As an exploratory analysis to assess group differences, each clinical outcome as continuous variables (ie, length of hospital stay and the number of comorbidities) and categorical variables (ie, hospital readmission) is analyzed using 2-sample *t* tests for continuous variables and Fisher exact test will be used for analyzing categorical variables. An exploratory analysis to assess differences in the postintervention self-elected exercise for the waitlist control group will be assessed for end points of aim 1-2 using paired *t* tests and Cohen d is calculated to determine the effect size.

## Results

The trial was funded in 2022 (DFCI McGraw/Patterson Research Fund from the Division of Population Sciences at the DFCI)). As of October 2024, a total of 3 participants have consented and been randomized (n=1, exercise group; n=2, waitlist control group). Trial completion and start of data analysis is expected in July 2025 with expected results to be published in early winter of 2026.

## Discussion

### Expected Findings

The primary purpose of the PROTECT Trial is to investigate if an 8-week digitally supervised prehabilitative exercise intervention improves lower limb muscle strength in patients with multiple myeloma. The PROTECT Trial provides a comprehensive assessment of physical capacity, patient-reported health outcomes, and cardiometabolic health as secondary outcomes, and evaluates patient posttransplant outcomes as tertiary outcomes. Furthermore, we will examine the effects of the patient’s self-elected exercise intervention following the ASCT recovery period to explore the benefits of prehabilitative versus rehabilitative exercise.

Outcomes from the PROTECT Trial will provide vital information on muscle strength development using a prehabilitative exercise modality in a patient population with low health status. Various exercise modalities targeting muscle strength have been assessed across cancers, with the most highly studied in breast, prostate, and colorectal cancers, and few interventions implemented within patients with multiple myeloma.

The goal of the proposed exercise intervention in the PROTECT Trial is to improve muscular strength in patients with multiple myeloma, a population more advanced in age (ie, 31.2% aged 65-74 years) [52]. The development of sarcopenia, as a result of disease-related physical inactivity due to immobility or disability (ie, fatigue in response to induction therapy, bone pain as a result of lesions due to disease progression) [53], is associated with post-ASCT complications in patients with multiple myeloma [7] and is indicated by decreased muscle strength [54]. Additionally, inflammation contributes to muscle wasting and may result from tumor cells releasing cytokines, though the biological mechanism of exercise and the inflammatory response is presently unclear in multiple myeloma [55,56]. Chronic low-grade inflammation, common in older adults (inflammaging), may also contribute to decreases in muscle mass, strength, and function (sarcopenia) through multiple pathways, primarily, mTORC1-mediated protein synthesis, metabolism, and mitochondrial biogenesis [56,57].

Proposed mechanisms for counteracting sarcopenia include reducing inflammation, promoting exercise-induced muscle hypertrophy, and decreasing fat infiltration following exercise (sarcopenic obesity) [58-60]. In fact, exercise training attenuates protein degradation signaling pathways and stimulates muscle protein synthesis, with overall anti-inflammatory and antioxidative effects that can mitigate both the sarcopenic effects and cancer-induced muscle wasting that occur [61,62]. In older adults specifically, muscle mass and function are improved with exercise, which increases the expression of insulin-like growth factor 1 and the subsequent increase of muscle protein synthesis [63]. Improved muscular function and strength following aerobic and resistance exercise interventions have been demonstrated in healthy adults and hematologic oncology populations [12,15,64]. Taking these processes and clinical populations into account, we hypothesize the proposed prehabilitative aerobic and resistance exercise intervention will target muscle protein synthesis to improve muscle strength in patients with multiple myeloma scheduled for ASCT.

Current evidence suggests muscle hypertrophy occurs with an estimated number of 18 resistance training sessions, performed over 6-10 weeks [65], which will be captured in the eight-week training period of the proposed exercise intervention. Effective resistance training oriented for hypertrophy consists of mechanical tension and metabolic stress [66] and promotes muscle protein synthesis in relation to muscle protein degradation [67]. The combination of increased muscle protein synthesis, decreased muscle protein degradation, and overall motor learning are supportive of pathways at the molecular level that lead to increased muscle mass, increased muscle force production, and overall physical strength [67]. Furthermore, resistance exercise has been implicated in stimulating the mTORC1 pathway (master growth regulator that senses and integrates diverse nutritional and environmental cues including growth factors, energy levels, cellular stress, and amino acids), which may improve mitochondrial function, increase muscle mass, and improve metabolic health, all in support of the development of muscular strength [62]. Hence, applying said principles of exercise to patients with multiple myeloma supports the primary outcome measure, improving muscular strength, of the PROTECT Trial.

Mechanisms for improvements in physical capacity, cardiometabolic health outcomes, and patient-reported outcomes along with posttransplant clinical outcomes, are described below. Exercise, particularly resistance training as noted above, stimulates muscle protein synthesis, leading to increased muscle mass and strength. However, resistance training also has the potential to improve endurance by enhancing mitochondrial function and improving motor control by neuronal growth. Cardiovascular adaptations increase oxygen transport capacity, cardiovascular health, and metabolic regulation, mitigating the risk of chronic diseases of aging. Additionally, exercise provides mental health benefits, reducing stress and enhancing cognitive function, potentially prolonging longevity and enhancing quality of life, particularly in patients with cancer undergoing pre- and posttransplant therapy.

Prehabilitative exercise, or exercise prior to medical treatment, has been successfully deployed in presurgical candidates and other tumor sites, yet there is a paucity of evidence on the effect of prehabilitation in patients receiving transplant; IMPROVE-BMT is an ongoing investigation to address this topic [68]. A recent study shows that a multimodal intervention program with partially supervised exercise training combined with nutritional support prior to transplant is feasible and safe. Patients showed improvements in fat-free muscle mass, physical performance, and health-related quality of life [69]. Additional research is needed to assess outcomes that support quality of life, overall survival, and relevant clinical outcomes along with the challenges of delivering the exercise in hospital and outpatient settings. Importantly, recent investigations indicate the acceptability and feasibility of delivering exercise prehabilitation, in person and digitally within the ASCT pathway in myeloma, but warrant further investigation on the combined effects and at the level of personalized or individualized therapy [70].

The prehabilitation intervention investigated in this study represents a novel approach to addressing the limited evidence on the benefits of prehabilitation. Prehabilitation, broadly recognized for its potential to enhance physical and psychological resilience, holds promise in optimizing outcomes for patients with cancer. Specifically, its application in the context of multiple myeloma remains understudied. The exploratory aims intend to examine the comparative effectiveness of rehabilitation versus prehabilitation, providing valuable insights into their respective contributions to patient well-being in this clinical setting.

The design of this trial includes digitally supervised, individually tailored prehabilitative exercise intervention that is progressed over the course of 8 weeks. Digital supervision allows for participants who are immunocompromised to safely complete the exercise at home and reduces the need to travel to and from the study site, outside of testing visits that are coordinated with ongoing treatment plans. Each program is designed to progress each week at 5% increments based on baseline strength and cardiorespiratory measures. The individually tailored aspect provides flexibility for patients who experience treatment-related symptoms or are able to easily manage the prescribed exercise.

### Strengths and Limitations

Strengths of this study design include a rigorously designed exercise intervention, taking into account exercise tolerance, bone pain, development of bone lesions, and limited mobility, and is individually tailored and supervised by a qualified exercise trainer. This approach supports gradual increases in load and volume over the training period and enhances program adherence. The training is conducted digitally, allowing individuals who cannot travel to the research site three times per week to participate and provides scheduling flexibility.

Limitations of this study design include a limited sample size, digital delivery, and short duration of exercise. Training is conducted remotely via a video communication platform Zoom (Zoom Video Communications, Inc), which may restrict the assessment of the full range of motion and utilization of alternative techniques to ensure the correct execution of each movement. Additionally, digital training limits the ability to assess the participant’s state through breathing and facial expressions, potentially impacting the reported RPE and the ability to monitor progression accurately.

### Conclusions

The PROTECT Trial will contribute to understanding the role of prehabilitative exercise, in a digitally supervised setting, on muscular strength outcomes in patients with multiple myeloma who receive ASCT. Findings from this trial will provide information on patient health outcomes following transplant including overall functional capacity. Understanding the limitations to exercise within this population may help develop future larger-scale exercise interventions in the prehabilitative and rehabilitative spaces that support patients with multiple myeloma from the time of diagnosis into survivorship.

## Abbreviations

ASCT: autologous stem cell transplant
DEXA: dual-energy x-ray absorptiometry
HR: heart rate
PROTECT: Prehabilitation Exercise Training in Multiple Myeloma Patients Undergoing Autologous Stem Cell Transplantation
REDCap: Research Electronic Data Capture
RM: repetition maximum
RPE: rating of perceived exertion

## Data Availability

Data sharing is not applicable to this paper as no datasets were generated or analyzed during this study.
